# SPR Sensor Based on a Tapered Optical Fiber with a Low Refractive Index Liquid Crystal Cladding and Bimetallic Ag–Au Layers

**DOI:** 10.3390/s22197192

**Published:** 2022-09-22

**Authors:** Joanna Korec, Karol A. Stasiewicz, Leszek R. Jaroszewicz

**Affiliations:** Institute of Applied Physics, Military University of Technology, 2 Kaliskiego St., 00-908 Warsaw, Poland

**Keywords:** surface plasmon resonance, optical fiber taper sensor, liquid crystal device, bimetallic layer, gold, silver

## Abstract

This paper presents a study of the influence of bimetallic layer covers of a tapered optical fiber surrounded by a low refractive index liquid crystal on the properties of light propagation in the taper structure. This research follows previous works on the effect of monometallic thin films (Au and Ag). In this case, the total thicknesses of the bimetallic layers were *h* = 10 nm, and the participation of gold and silver was equal. The films were deposited on one side of the tapered waist area. The liquid crystal cells were controlled with a voltage U from 0 to 200 V, with and without amplitude modulation at a frequency of *f_mod_* = 5 Hz. For the purposes of this research, spectral characteristics were obtained for a wavelength λ ranging from 550 to 1200 nm. Measurements were carried out at room temperature for three types of rubbed layers orientation—orthogonal, parallel, and twist in relation to the fiber axis. Obtained resonant peaks were compared with the previous results regarding the resonant wavelength, peak width, SNR, and maximum absorption. In the presented paper, the novelty is mainly focused on the materials used and their time stability, as well as corresponding changes in the technological parameters used.

## 1. Introduction

Sensing techniques based on optical fibers have been known since 1967, when the first Fotonic sensor was patented [1,2]. Over the years, sensors have advanced together with improving optical fiber technology. The surface plasmon resonance effect generated on the optical fiber elements is a relatively new sensory technique. It has been known since 1990, when Villeuendas and Pelayo introduced an SPR sensor with a modified optical fiber head [3]. The optical fiber probe is a modification of the traditional Kretschmann configuration, but the role of the prism is taken over by the optical fiber core [4]. As it is well known, direct light cannot excite the surface plasmons but an evanescence wave (EW) [5]. In an optical fiber without changes in the structure and geometry, EW has too low a penetration depth for interaction with the external medium [6,7]. Hence, some changes are necessary. Regarding the modification site on the optical fiber, probes can be divided into side surface modified and forehead modified [8]. Among the side adjustments, there are few manufacturing methods: etching [9] or polishing [10] D-shape fiber formation or heterocore [11], bending (u-bent) [12], cavity [13], and tapering [14]. The forehead modification can be distinguished by tips, especially mirrors [15], angle polishing [15], tip tapering [16], and LRPR tip [17]. Based on recent computer simulations covering MOF holes can improve the properties of SPR optical fiber probes [18,19]. All these changes lead to the enhancement of the EW wave vector and have a direct influence on the dynamic range of the sensor, its sensitivity, and its accuracy. The degree of coupling between plasmons and EW highly depends on wavelength, metal layer thickness, as well as on the type of optical fiber [20].

Plasmonic materials can be deposited on the optical fiber probe surface using different physical and chemical methods. The physical ones (e.g., thermal vapor deposition, sputtering) are mostly characterized by directional deposition of materials, and the formed layers are asymmetric. To obtain even layers around cylindrical surfaces, rotating special units are needed. Therefore, chemical methods, including chemical vapor deposition [21] and currentless [18] deposition are better solutions allowing coverage of cylindrical surfaces, including inside PCF holes [18,22]. 

In the case of SPR probes manufacturing, three types of coverage can be highlighted as asymmetric: one-sided and symmetric: two-sided (thicker cover on the upper and bottom; thinner cover on the sides) and cylindrical (uniform thickness around the surface). Both covers: one-sided and two-sided, increase the dynamic range of the sensor and simultaneously can contribute to the widening of resonant dips [23]. Better results are obtained for cylindrical covers [24]. The SPR effect is achieved in metals because they are characterized by the complex dielectric constant *ε_m_*, which can be expressed as *ε_m_ ≡ ε_r_ + iε_i_* (where *ε_r_* is the real part related to reflection and ε_*i*_ is the imaginary part of the dielectric constant related to losses). SPR spectrum is very sensitive to both parts; hence, the width and depth are related to the ratio of *ε_r_/ε_i_* [25]. As this ratio increases, the narrower and deeper the resonant dip [26]. Due to low chemical reactivity, noble metals like Au, Ag, Pd, or Pt are mostly used for coating purposes [27,28]. Recently, the additional layers, such as oxides TiO_2_, ZnO, ITO, In_2_O_3_, Ta_2_O_5_, and graphene, are gaining more and more popularity [29,30]. This is because these materials enhance sensitivity and accuracy, and provide long-term stability, as well [31,32].

This paper presents an SPR probe using a tapered optical fiber (TOF) covered with bimetallic layers Au–Ag and Ag–Au. Tapers, due to their characteristic geometry and optical properties, allow the construction of hybrid structures using liquid crystal (LC) as a sensing medium, whose range of effective refractive index (RI) can be controlled by an electric field, temperature, or magnetic field. The created system contains two electrodes permanently connected to a liquid crystal cell (LCC). Inside this cell, a tapered fiber and LC are placed. If the LCC is thin enough, it is possible to steer the arrangement of LC molecules by using an electric field. Consequently, sensing of changes in the external environment is obtained by changes in the effective RI inside the medium, as was proved in the previous papers [14,33]. The resonant dips occur when no electric field is applied. The application of an additional current source causes changes in the effective RI and boundary conditions of the propagated light inside the taper; hence, resonant dip shifts are observed. This research is an extension of the previous works about the influence of monometallic layers: gold and silver. As it was observed, LCC, which consisted of only a single metal cover, had several disadvantages from a sensory point of view, e.g., wide resonant dips, high intermodal interference, and low SNR. Based on the literature, the combination of these two noble metals should improve some of these issues; thus, it was decided to combine them into two bimetallic films: Au/Ag and Ag/Au [26,34]. The novelty is based on the covers used, created from these metals, and the order in which they are deposited on the tapered fiber surface. In the present work, the comparison of the received resonant dips and results obtained for LCC with gold is provided. Moreover, this paper contains the summation of all the results obtained so far.

## 2. Materials and Methods

**Figure 1:**
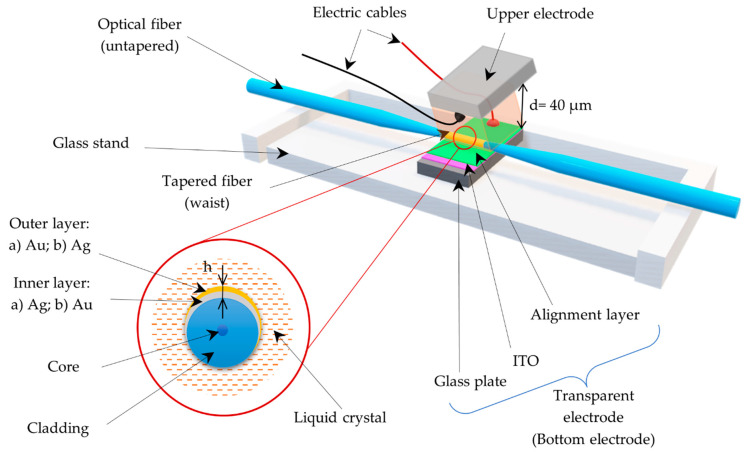
Scheme of the LCC probe with the bimetallic cover.

For the purpose of this work, the tapers were manufactured using a single-mode optical fiber for a telecommunication range. The manufacturing system FOTET (Fiber Optic Taper Element Technology) is a dedicated device for manufacturing tapered optical fiber by the flame brush method, using a low pressure burning powered by a propane-butane-oxygen gas mixture. System FOTET is controlled by computer software which allows the selection of the appropriate parameters of elongation, e.g., length and diameter of the manufactured taper. The full process description is included in our previous work [33]. The formed LCC contains a tapered optical fiber with a tapered waist area of approx. 5 mm. The obtained optical fiber tapers were characterized by the following parameters: length l = 20.0 ± 0 4 mm with a diameter of the tapered area *φ* = 15 µm and attenuation below α = 0.3 dB at 1550 nm for single-mode fiber. TOF parameters were experimentally selected to obtain thin LCCs and reduce the applied LC steering voltage, as well as the highest sensitivity and influence of the deposited films. The thickness of the created LCC is maintained by spacers with a diameter, *d* = 40 µm. The bimetallic layers, Ag/Au and Au/Ag, were deposited on the surface of the TOF in a two-stage sputtering process. Regarding the type of cover, the inner film was gold or silver. The thickness of the deposited bimetallic layers was considered from the existing literature [35] for gold layers and [36,37] for silver layers. Based on the previous works, the total thickness of the bimetallic layers was estimated as *h* = 10 nm, and the contribution of the individual metals was 50% because the probes that the multilayer covers work better with thinner silver layers [38]. Sputtering was provided by using an EM SCD500 (Leica, Wetzlar, Germany) sputtering coater. The pressure inside the chamber was over 10^−2^ mbar and the thickness of the deposited layer was continuously measured by using QCM. Figure 1 presents the scheme of the LCC with the TOF.

## 3. Results and Discussion

**Figure 2:**
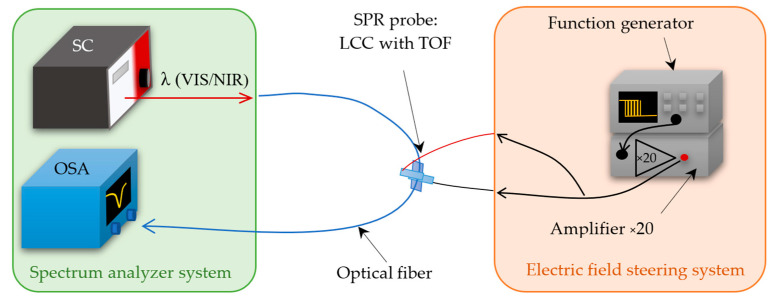
Scheme of the measuring system containing the spectrum analysis unit and the part responsible for controlling the LC molecules arrangement inside the LCC, as well.

Manufactured liquid crystal cells were examined using a measuring system that contained supercontinuum broadband light source SuperK EXTREME (NKT Photonics, Birkerød, Denmark), which operates at the wavelength range of VIS-NIR (400–2400 nm) and an OSA AQ6373B optical spectrum analyzer (Yokogawa, Tokyo, Japan) with a detection range of 350–1200 nm, in addition, to a DG1022Z function generator (RIGOL, Beijing, China) together with the amplifier. The last part of the system is responsible for controlling liquid crystal molecules inside the cell by generating an electric field. Figure 2 (below) presents the measurement system used, which is divided into two sections: Spectrum Analyzer System with SC and OSA and Electric Field Steering System with a function generator and amplifier.

### 3.1. TOFs Covered with Bimetallic Au/Ag Film

**Figure 3:**
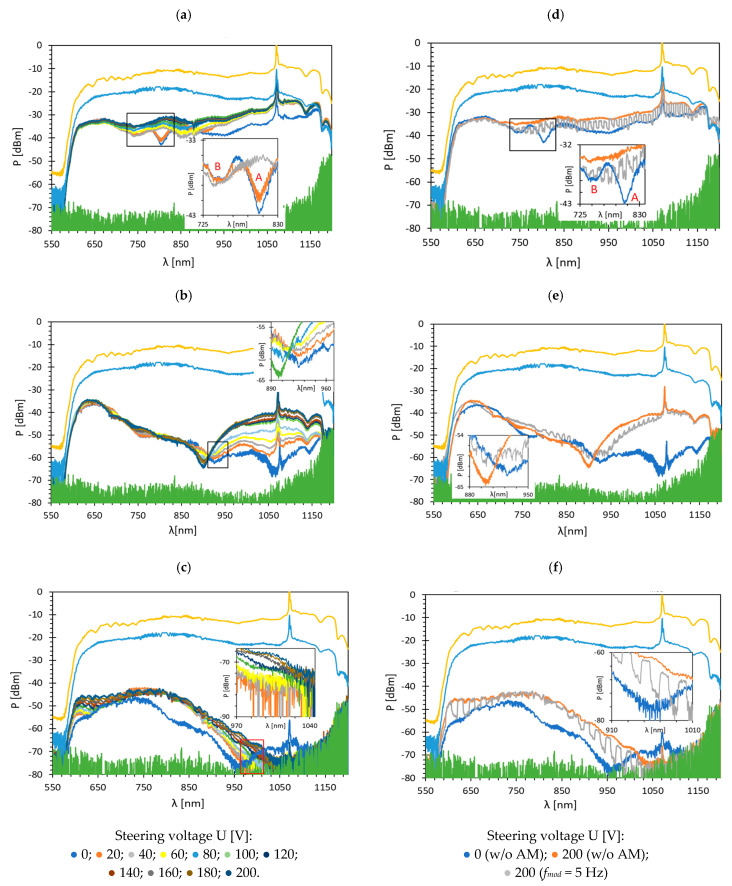
Spectra obtained for TOF with bimetallic Au/Ag layer coating for: (**a**) orthogonal; (**b**) parallel; (**c**) twisted cell under voltage U: 0–200 V w/o AM, and dynamic response of: (**d**) orthogonal; (**e**) parallel; (**f**) twist cell with *f_mod_* = 5 Hz with 100% AM.

Measurements were carried out at room temperature for three types of LC cells: orthogonal (⊥), parallel (∥), and twist (⊥/∥). Such types of LCs are formed through the appropriate arrangement of the transparent electrodes (with the rubbed surface) with respect to each other and the taper axis. The voltage range used to switch LC equaled U from 0 to 200 V without (w/o) amplitude modulation (AM) and with a 100% AM depth at a frequency of *f_mod_* = 5 Hz. Such voltage allows reorienting the entire volume of LC inside the LCC. Figure 3 presents transmission spectra through the LCC with TOF covered with a bimetallic Au/Ag layer for the wavelength range *λ*: 550–1200 nm. The chosen wavelength range has to do with the transmission properties of TOF, especially the penetration depth of the beam and resulting losses. In all pictures, the reference measurement performed for TOF w/o a metallic layer is characterized by the lowest attenuation (the highest transmission) and is marked in yellow. The blue color corresponds to the transmission in a TOF covered with a metallic layer measured in the air (w/o LC inside the cell). The lowest transmission (green) is the noise level in OSA. Figure 3a–c presents the results obtained without AM and Figure 3d–f presents the dynamic response obtained for AM *f_mod_* = 5 Hz.

Based on the data obtained for TOF w/o metallic film measured in air, and TOF with the cover (also in the air), it can be observed that the bimetallic cover of Au/Ag causes attenuation with a value similar to the silver layers [39]. As before, the main dips (A) are shifted from the peaks obtained for single metallic layers and are equal to $\lambda_{res}^{⊥}=$ 803.6 nm, $\lambda_{res}^{∥}$ = 926.4 nm, and $\lambda_{res}^{⊥/∥}$ = 968.4 nm for orthogonal, parallel, and twist, respectively. In all cases, but most visible for the orthogonal cell, the secondary dip (B) appears for a shorter wavelength ($\lambda_{res\;B}^{⊥}=819\;\mathrm{nm})$ and close to the resonant dip. It can be found that the primary peak is sharper than the additional peak. As the electric field increases from 0 to 40 V, the additional peak becomes slightly deeper, and the primary peak becomes shallower. This may indicate the presence of strong coupling. Moreover, increasing the applied voltage causes a decrease in the coupling energy and weakens the SPR effect [40]. This phenomenon can be observed when the energy transfer rate between light and exciton is faster than their average dissipation. It results in the periodic character of the energy exchange and causes the formation of additional dips [41]. Moreover, as the electric field control increases, the transmission in the cell increases, as well. However, in the case of a parallel and twisted cell, this relation is more visible for longer wavelengths. As can be noticed for a bimetallic film, the orthogonal cell is characterized by the highest power level. In the dynamic response graphs (Figure 3d–f), it can be observed that on/off switching of the cell does not occur in the full wavelength range, and spectra obtained for U = 200 V with AM *f_mod_* = 5 Hz are narrower relative to the changes in power levels between U = 200 and U = 0 V w/o AM. The possible explanation of this phenomenon is the relatively long response time of the LC used due to its low dielectric anisotropy [42].

**Figure 4:**
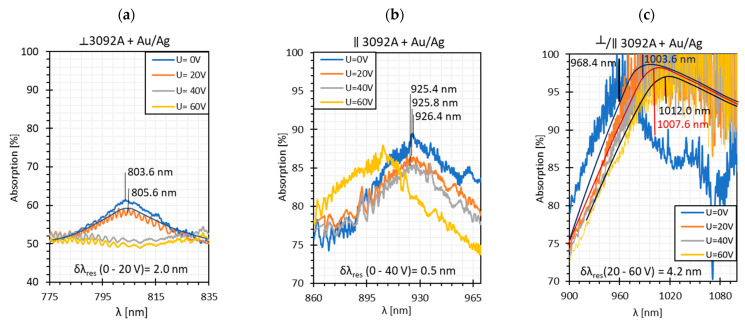
Absorption peaks obtained for (**a**) orthogonal, (**b**) parallel, and (**c**) twisted cells. The solid lines are the fitting curves obtained for absorption plots.

Based on the obtained transmission spectra, absorption plots, along with resonant resolutions *δλ_res_* for all LC cells, were determined. Figure 4 presents the obtained results. The average noise power level of −75 dBm was taken as 100% absorption. As can be noticed, the highest absorption peaks occur for twisted and parallel cells and are equal to 100% and 90%, respectively. In the case of the orthogonal cell, the absorption is the lowest and is approximately 60%. High power fluctuations are visible on the presented relations, but they are slightly lower than in the case of the single metal cover [14,39]. This is probably due to the deposition of a thinner metallic film, in particular the silver one (*h_Ag_* ~ 5 nm). As can be observed, the peaks obtained for the twisted cell are the most “deformed” and are at the limit of detection and noise level.

Because of high intermodal interference, the resonant resolutions have been approximated. For the orthogonal cell, the *δλ_res_* = 2.0 nm was obtained for U in the range from 0 to 20 V, and this value is similar to the results obtained for pure gold and pure silver films: $\delta \lambda_{res}^{Au}=2.35\;\mathrm{nm}\;\mathrm{and}\;\delta \lambda_{res}^{Ag}=2.6\;\mathrm{nm}$ [14,39]. In the case of the parallel cell, the resonant resolution was calculated for U: 0–40 V and amounts to *δλ_res_* = 0.5 nm. This value is close to the resolution obtained for the silver cover, $\delta \lambda_{res}^{Ag}=0.4\;\mathrm{nm}$. As was mentioned before, for the twisted cell, the peaks are highly approximated, as well as the established parameter *δλ_res_* = 4.2 nm, as well.

### 3.2. TOFs Covered with Bimetallic Ag/Au Film

**Figure 5:**
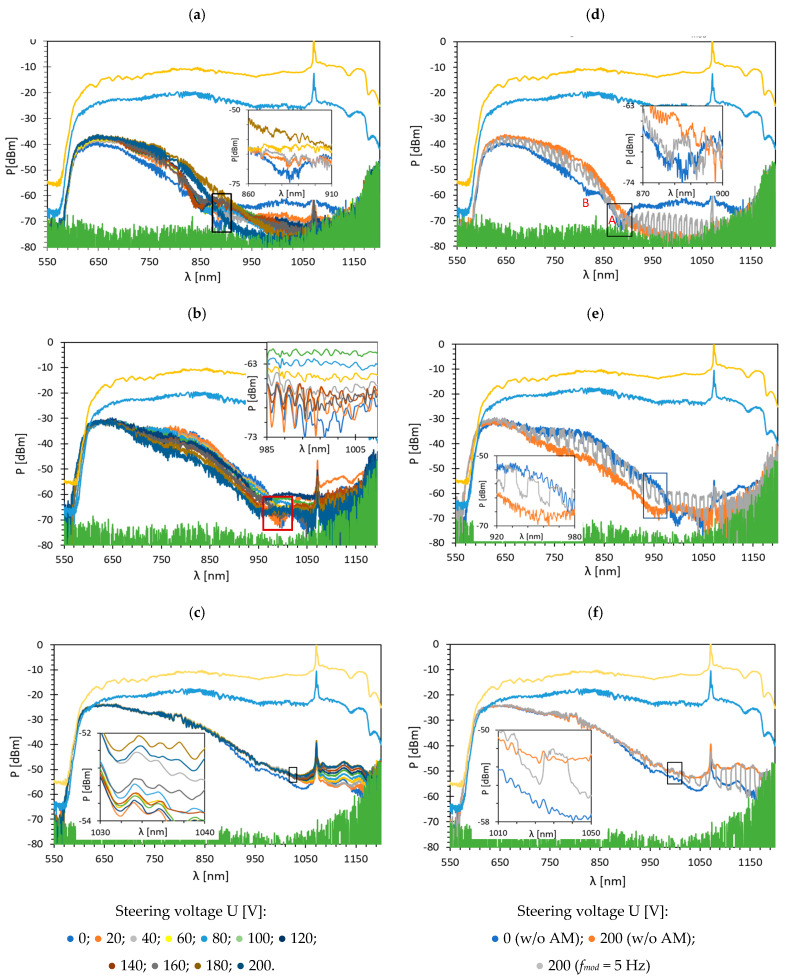
Spectra obtained for TOF with bimetallic Ag/Au layer coating for: (**a**) orthogonal; (**b**) parallel; (**c**) twisted cell under voltage U: 0–200 V w/o AM, and dynamic response of: (**d**) orthogonal; (**e**) parallel; (**f**) twist cell with *f_mod_* = 5 Hz with 100% AM.

The second configuration is a combination of Ag/Au layers; all deposition parameters were the same as in the previous case. Total thickness was equal to *h* = 10 nm with a layer thickness of 50% Ag and 50% Au. Figure 5 presents the results obtained for the wavelength range of 550–1200 nm. As can be observed in the figure, for measurement in air, the attenuation caused by a bimetallic Ag/Au film is almost the same as for an Au/Ag film and is approximately −10 dBm. The main difference between the obtained transmissions for Ag/Au and Au/Ag is the shape of the spectra, where the lowest power level is observed in the NIR region. This is caused by the silver layer, which is the first one on the TOF side and absorbs part of the light beam. Such a trend is also observed in the cell with pure silver layer covers [39]. Graphs in Figure 5a–c present spectra for the steering voltage *U*: 0–200 V w/o AM and in Figure 5d–f for 100% AM with *f_mod_* = 5 Hz. An additional difference between both bimetallic covers is that for the Ag/Au film, the dips are obtained only for two LC cell types: orthogonal and parallel. For twist resonant peak was not observed. 

Their resonant wavelengths are equal to $\lambda_{res}^{⊥}$ = 886.3 nm and $\lambda_{res}^{∥}=$996.4 nm, respectively, and they are moved by approximately 214.1 nm relative to the dips obtained for LCC with pure gold and 155 nm relative to the dips obtained for LCC with pure silver. Based on these shifts, a possible dip for the twisted cell could occur at 1050 nm, where the power reduction is observed, however, without the formation of a peak. For this metallic cover, as in the former case, the additional dips occur when the electric field is not applied. It should be noted that the difference between the main and the shallower dip is similar to that of the orthogonal cells with Au/Ag and is approximately 67 nm.

**Figure 6:**
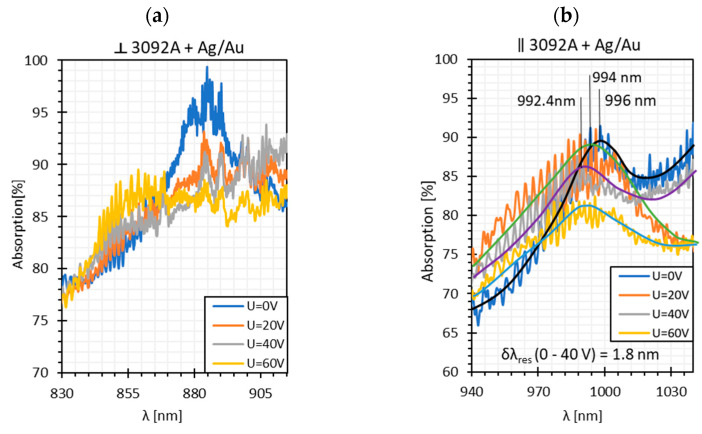
Absorption plots were obtained for (**a**) orthogonal and (**b**) parallel cells. The solid lines (black, blue, green, and purple) are the fitting curves obtained for the absorption plots.

Among all LC types, the twisted cell is characterized by the highest transmission; however, its dynamic response is very narrow and differences in power levels between on/off states are negligible at the visible wavelength range. The other two LC cells have a similar dynamic response range and transmission for shorter wavelengths, but it changes in the NIR/IR region, and the orthogonal cell indicates significantly higher losses. Based on these spectra, the absorption plots were calculated and presented in Figure 6.

As can be noticed, high fluctuations occur; hence, the estimation of *δλ_res_* was difficult. In the case of the orthogonal cell, this parameter was not determined and for the parallel cell, it was estimated to be $\delta \lambda_{res}^{∥}=$ 1.8 nm. This is the highest value of *δλ_re_*_s_ obtained for parallel cells among all metallic covers used.

The above results were used to estimate the differences in the obtained resonant dips. Table 1 presents the previously obtained resonant dips for LC cells with the gold layer and these data were used to compare dips obtained for bimetallic covers. The following parameters were considered first:$\;\lambda {\;}_{res}^{\left(0V\right)}$—resonant wavelength (for U = 0 V); $\lambda_{0.5}^{\left(0V\right)}$—resonant dip width measured at 0.5 peak height, quality factor *Q* (is a ratio of $\lambda {\;}_{res}^{\left(0V\right)}\;\mathrm{and}\;\lambda_{0.5}^{\left(0V\right)}$), and the SNR. Moreover, the additional factors were estimated: $\Delta \lambda_{res}^{x-Au}$—resonant dip shift between the peak of the chosen bimetallic layer (where *x =* Au/Ag or Ag/Au) and Au, as well as $\Delta \lambda_{0.5}^{x-Au}$—peak width difference between x and Au was calculated. As before, for orthogonal and twisted cells, the dips obtained without applied electric field (U = 0 V) are compared, and for parallel cells, the peaks obtained for U = 0 V and U = 200 V are taken into consideration. Thus, an additional parameter such as $\Delta \lambda_{res}^{\left(0-200\;V\right)}$—the difference between peaks obtained for 0 and 200 V—and $d\lambda_{0.5}^{\left(0-200V\right)}$—the difference in width of peaks obtained for 0 and 200 V—were estimated. The results in Table 2 and Table 3 present a comparison of resonant dips of orthogonal, twisted, and parallel cells compared to the dips obtained for cells with gold films. 

Comparing results (obtained only for U = 0 V) from Table 2 and Table 3, it can be observed that the resonant peaks obtained for probes with Au/Ag and Ag/Au layers have shifted approximately about 138 nm and slightly above 214 nm, respectively. In the case of the parallel cells, the additional parameter $d\lambda_{res}^{\left(0-200\;V\right)}$ shows that for both cases, $d\lambda_{res\;\left(Au-Ag\right)}^{\left(0-200\;V\right)}$ and $d\lambda_{res\;\left(Ag-Au\right)}^{\left(0-200\;V\right)}$, the difference between their peaks *λ*(0 V) and *λ*(200 V) is similar and is equal to approximately 37 nm.

Moreover, the distances between the peaks are comparable with those obtained for monometallic layers. The subsequent parameter under consideration is a peak width. In the case of probes with an Au/Ag layer, the width of the orthogonal and parallel cell is almost the same and equals 24 m. As before, for both covers, the width of resonant peaks measured at 0.5 height decreases for dips obtained without an applied electric field. Only in one case is the widening of dip observed—for a parallel cell with an Ag/Au layer at U = 200 V. The maximum reduction was obtained for the orthogonal cell covered with Ag/Au and amounted to 71.4%. What is interesting is the calculated difference, $d\lambda_{0.5}^{\left(200-0V\right)}$, for the parallel cell shows that an increase in the electric field also causes an increase in the peak width. For both cases, the enlargement amounted to approximately 6 nm. Regarding the SNR, this is the last parameter to be compared, and it was estimated using data from Figure 4 and Figure 6 and Table 2 and Table 3. For all LCC types with Au/Ag film, the SNR value was higher than for LCC with gold films; however, for LCC with Ag/Au, it was only possible to estimate SNR for the parallel cell.

**Figure 7:**
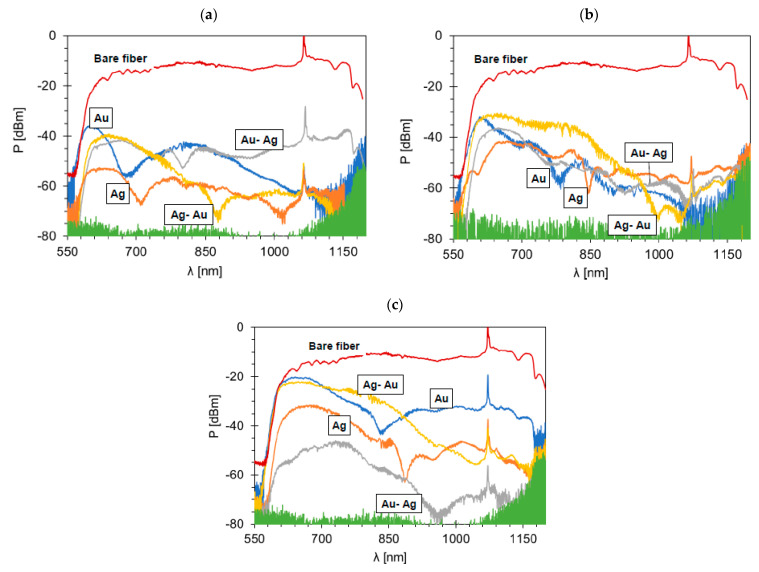
SPR dips were obtained for: (**a**) orthogonal; (**b**) parallel; (**c**) twisted cells with different metallic covers.

To compare the obtained resonant dips with the previous ones, Figure 7 summarizes all previous results for mono- and bimetallic covers without an applied electric field. The LCC responses for the individual metal cover are divided according to the LC cell type: orthogonal, parallel, and twist. First, it can be noticed that the twisted cell has the highest transmission among all LCC types regardless of the metallic film type. Based on the resonant wavelength location, it is likely that this LC cell has the highest effective RI among other cell types. The LC used, named 3092A, is a mixture of four substrates, and one of the components, whose concentration in the solution is 30%, is characterized by a negative dielectric anisotropy. Furthermore, this mixture does not consist of any cyano-compound, due to which the ordinary RI is very low, lower than the RI of the fiber.

The LC mixture contains only carbonates which causes the decrease in both crossing point temperature and RI. This makes it possible to obtain the SPR effect; however, it has been proved that a solution with an RI similar to the RI of the fiber cause intermodal interference to occur. Additionally, the lack of cyan compounds results in longer switching on/off times. SPR probes using gold or silver covers provide the resonant wavelengths in the visible wavelength range; hence, all peaks which appear at the limit of detection between the visible and the NIR region are highly deformed and indistinct. Based on the previous results from [14,39], the average shifts of the resonant dips were calculated. Differences were estimated with respect to the position of resonant peaks obtained for gold films, and it is the average value from all LC cell types for the selected cover. Additionally, only measurements without an electric field were under consideration. The estimated values are as follows: $\Delta \lambda_{res}^{Ag}=56.4\;\mathrm{nm};\;\Delta \lambda_{res}^{Au/Ag}=138.4\;\mathrm{nm};\;\Delta \lambda_{res}^{Ag/Au}=214.4\;\mathrm{nm}$. As for the width of resonance dips, the widest peaks are obtained for cells with gold films, and the highest value was obtained for LCC ⊥Au, which equaled $\Delta \lambda_{0.5}^{Au}=64.3\;\mathrm{nm}.$ The deposition of silver layers contributes to reducing the width of these dips. In the case of the orthogonal and parallel cells, the reduction was at least 50% in width for LCC with Au cover. However, the highest value was obtained for LCC ⊥Ag/Au, with a reduction of 71.4%.

The last parameters compared are SNR and absorption. Regarding the SNR, the best results were achieved for twisted and orthogonal cells covered with the Ag layer and were 0.336 and 0.110, respectively. Other results do not exceed the value of 0.100. These probes are characterized by high light absorption, and except for three cases (Au: ⊥ and ⊥/∥, and Au/Ag⊥ cells), the value reaches 90% and more. It should be mentioned that high absorption peaks can be caused not only by meeting the resonant conditions but also by the absorption of some of the optical power by deposited covers. As can be observed, the general power levels in most LC cells are rather low and highly dependent on the wavelength.

In the case of all parallel LC cells and two additional parameters, $d\lambda_{res}^{\left(0-200V\right)}$ and $d\lambda_{0.5}^{\left(200-0V\right)},$ it can be observed that the average distance between dips obtained for 0 and 200 V equals 37.4 nm; however, in the case of cells where Au is the mono cover or is the inner layer, this distance is lower than in the opposite case (with Ag mono- and inner layers). Moreover, the application of an external electric field narrows the width of the dip. The highest reduction is observed for bimetallic covers, where it was approximately 6 nm.

## 4. Conclusions

The performed research on the influence of depositing thin bimetallic layers on the tapered and confined optical fiber surface inside an LC cell allowed for the following conclusions to be drawn:

1. Resonant peaks obtained for Au/Ag cover are shifted by approx. 138 nm and 214 nm for Ag/Au compared to the peaks obtained for gold layers. Additionally, no resonant peak is observed in the case of the twisted cell covered with Ag/Au.
2. For both bimetallic covers, the highest reduction in the peak width occurs for orthogonal cells. For parallel cells covered with Ag/Au film, as an electric field increases, the dip width also increases.
3. Regarding the SNR, it is difficult to find any relation between value of the SNR and the LCC type or cover, but the highest values of the SNR were obtained for orthogonal and twisted Au/Ag cells, while for Ag/Au, these parameters could not be estimated. The SNR of the parallel cell with Ag/Au is three times higher than the same type of cell with the Ag/Au layer.
4. The level of absorption peak in each case is very high and, excluding the results obtained for the orthogonal cell covered with Au/Ag, reaches at least 90%.

Regarding all performed measurements for films: Au, Ag, Au/Ag, and Ag/Au, it can be concluded that the resonant wavelength highly depends on the metal that directly interacts with the light beam. The LCC where the silver films were used as an inner layer resulted in a larger shift in the dips compared to the gold layers. Moreover, these cells, in most cases, are characterized by narrower resonant dips and higher values of the SNR. As can be observed, the type of LCC influences power transmission. It can be stated that the twisted cells are characterized by the lowest attenuation among all probes.

This paper shows that only some of the measured parameters of the constructed system improved over the LCC covered with gold. Moreover, it was not possible to eliminate problems and add effects such as intermodal interference. The probes still require further investigation to use these structures in real applications. It is possible that additional layers like graphene, metal oxides, or other optical fibers would allow increasing the difference in the RI between the fiber and LC, potentially improving the results.

## Figures and Tables

**Table 1:** Resonant dips obtained for LC cells with the gold monolayers [14].

**LCC**	$\lambda {\;}_{res}^{\left(0V\right)}\;$	$\lambda_{res}^{\left(200V\right)}\;$	$d\lambda_{res}^{\left(0-200\;V\right)}\;$	$\lambda_{0.5}^{\left(0V\right)}\;$	$\lambda_{0.5}^{\left(200V\right)}\;$	$d\lambda_{0.5}^{\left(200-0V\right)}\;$	$Q_{\left(U=0V\right)}\;$	$Q_{\left(U=200V\right)}\;$	*SNR*
Orthogonal	665.8 nm	-	-	64.3 nm	-	-	10	-	0.036
Twist	831.4 nm	-	-	47.6 nm	-	-	17	-	0.076
Parallel	786.0 nm	749.7 nm	36.3	28.9 nm	31.9 nm	3 nm	27	23	- *

* Parameter could not be estimated because of high intermodal interference.

**Table 2:** Comparison of resonant dips of orthogonal and twisted cells compared to the dips obtained for cells with gold films.

Orthogonal Cell			Twisted Cell	
	Au/Ag	Ag/Au	Au/Ag	Ag/Au
$\lambda_{res}^{\left(0V\right)}$	803.6 nm	883.6 nm	968.4 nm	-
${\Delta \lambda }_{res}^{x-Au}$	137.8 nm	217.8 nm	137.0 nm	-
$\lambda_{0.5}^{\left(0V\right)}$	24.6 nm	18.4 nm	44.4 nm	-
${\Delta \lambda }_{0.5}^{x-Au\;*}$	−39.7 nm → −61.7%	−45.9 nm → −71.4%	3.2 nm → −6.7%	-
Resonant dip **	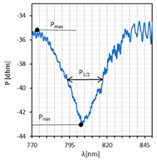	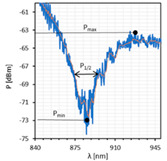	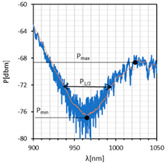	-
$Q_{\left(U=0V\right)}$	33	48	22	
SNR	0.081	0.000	0.095 ***	-

* Reduction in peak width relative to the Au layer; ** The solid lines are the fitting curves, *** Approximated parameter.

**Table 3:** Comparison of resonant dips of a parallel cell.

Parallel Cell		
	Au/Ag	Ag/Au
$\lambda_{res}^{\left(0V\right)}$ $\lambda_{res}^{\left(200V\right)}$ ${d\lambda }_{res}^{\left(0-200\;V\right)}$	926.4 nm 890.0 nm 36.4 nm	996.0 nm 957.8 nm 38.5 nm
${\Delta \lambda }_{res}^{x-Au\;\left(0V\right)}$ ${\Delta \lambda }_{res}^{x-Au\;\left(200V\right)}$	140.4 nm 140.3 nm	210.0 nm 208.1 nm
$\lambda_{0.5}^{\left(0V\right)}$ $\lambda_{0.5}^{\left(200V\right)}$ ${d\lambda }_{0.5}^{\left(200-0V\right)}$	24.0 nm 30.0 nm 6 nm	27.1 nm 33.6 nm 6.5 nm
$\Delta \lambda_{0.5}^{x-Au\;\left(0V\right)\;*}$ $\Delta \lambda_{0.5}^{x-Au\;\left(200V\right)\;*}$	−4.9 nm → −16.9% −1.9 nm → −5.9%	−1.9 nm → −6.6% +1.7 nm → +5.3%
Resonant dips **	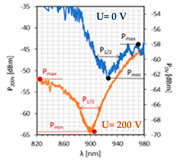	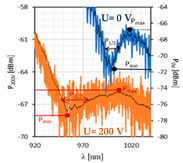
$Q_{\left(U=0V\right)}$ $Q_{\left(U=200V\right)}$	39 30	37 29
SNR	0.021	0.066 ***

* Reduction in peak width relative to the Au layer; ** The solid lines are the fitting curves, *** Approximated parameter.
